# Comparative evaluation of Bacillus and Lactobacillus in cottonseed hull fermentation

**DOI:** 10.1186/s12866-026-05090-9

**Published:** 2026-08-05

**Authors:** Kunyi Li, Yang Li, Xin Huang, Huaibing Yao, Gulinigaer Aiyisirehong, Nuerbiya Aibaidula, Weidong Cui, Min Hou

**Affiliations:** 1 Xinjiang Laboratory of Special Environmental Microbiology, Institute of Microbiology, Xinjiang Academy of Agricultural Sciences, Urumqi, 830091 China; 2 Key Laboratory of Biological Resources and Genetic Engineering, College of Life Science and Technology, Xinjiang University, Urumqi, 830017 China

**Keywords:** Cottonseed hulls, Microbial community dynamics, Nutrient transformation, Correlation network

## Abstract

Microbial fermentation is an effective approach to improving the nutritional value of cottonseed hulls and alleviating feed resource shortages. However, the interaction mechanisms between *Bacillus* and *Lactobacillus* within fermentation systems remain unclear. In this study, a composite *Bacillus* group (Group B), a composite *Lactobacillus* group (Group R), and a natural fermentation control group (Group C) were established for a 30-day solid-state fermentation experiment. Physicochemical parameters were measured at multiple time points, and 16 S rRNA amplicon sequencing was performed to systematically analyze fermentation quality changes and microbial community succession. It was found that, compared with Groups R and C, Group B exhibited superior performance in degrading antinutritional factors and macromolecular substrates. The degradation rate of free gossypol reached 80.52%, while crude fiber content was reduced by 14.98%. Microbial community structure analysis revealed significant differences in composition among the treatment groups. Functional prediction analysis suggested that Group B was enriched in pathways associated with carbohydrate and amino acid metabolism, whereas Group R showed higher representation of pathways related to energy metabolism and cofactor and vitamin biosynthesis. Network analysis demonstrated that the introduction of *Bacillus* increased microbial network complexity while reducing cooperative interactions, indicating a strong competitive effect during fermentation. LEfSe analysis further revealed that different treatments significantly shaped distinct microbial biomarkers: Group B was enriched in *Acinetobacter*, *Brevibacterium*, and *Faecalibacterium*; Group R was dominated by *Acidobacterium* and *Megasphaera*; and Group C was dominated by *Enterobacter* and *Staphylococcus*. Correlation analysis revealed that 19 bacterial genera were significantly associated with nutrient transformation indicators, suggesting a strong coupling between microbial structure and functional processes. In summary, *Bacillus* and *Lactobacillus* exhibit a functional division of labor, characterized by early-stage degradation and later-stage stabilization during cottonseed hull fermentation. This study provides a theoretical foundation for optimizing microbial consortia and promoting the high-value utilization of agricultural waste.

**Supplementary Information:**

The online version contains supplementary material available at https://doi.org/10.1186/s12866-026-05090-9.

## Introduction

With the sustainable development of global agriculture and animal husbandry, agricultural processing by-products have emerged as a critical means of addressing the shortage of feed resources. Cottonseed hulls, a by-product of cotton processing, exhibit very low utilization rates owing to their high cellulose content and low nutritional value. Microbial solid-state fermentation is one of the safest and most effective methods for utilizing cottonseed hulls. It isproves their nutritional value by breaking down their lignocellulose and anti-nutritional components [1]. Solid-state fermentation enables microorganisms to be in close contact with the substrate, leading to a higher concentration of metabolic products(e.g., organic acids, hydrolytic enzym-es, bacteriocins) [2, 3]. For instance, the protein content of Rhizopus biomass produced via solid-state fermentation has increased threefold compared to its initial value [4]. Furthermore, microbial solid-state fermentation utilizing agricultural waste (such as corn and weeds) can achieve a utilization rate exceeding 90% [5].

*Bacillus* and *Lactobacillus* have been identified as the key bacterial genera responsible for degrading free gossypol and enhancing the nutritional value of feed. *Bacillus* has been shown to significantly degrade gossypol while disrupting the structure of lignocellulose [6]. *Lactobacillus* has been found to effectively inhibit the proliferation of pathogenic bacteria while simultaneously breaking down large molecular proteins into small peptides and amino acids [7, 8]. However, during fermentation, the effects of inoculating various microorganisms on the microbial community vary significantly. Niu et al. [9] found that *Bacillus* may alter substrate availability by degrading lignocellulose, thereby inhibiting the growth of native organisms; Park et al. [10] indicated that although the metabolic products of *Lactobacillus* can optimize the proteolysis process, reversing the succession trend of the microbial community in the later stage of fermentation is challenging. Furthermore, Mawarda et al. [11] observed that exogenous *Bacillus* with strong resource utilization capabilities could alter the microbial structure of low-diversity soil. While *Lactobacillus* lowers the environmental pH by producing acid, they may selectively promote the proliferation of acid-tolerant bacteria [12]. These contradictory results suggest that the mechanisms of influence of different bacterial genera on microbial communities may be closely related to their functional specificity, substrate interactions, and niche competition [13, 14]. However, there is currently a lack of systematic comparisons of the dynamic changes in microbial communities of *Bacillus* and *Lactobacillus* during the fermentation process of cottonseed hulls, leading to an insufficient understanding of the action mechanisms and synergistic relationships of these two genera. Therefore, systematically comparing the dynamic changes in microbial diversity during the fermentation of cottonseed hulls by *Lactobacillus* and *Bacillus* holds significant scientific value for revealing the synergistic dynamics of these two genera and optimizing the degradation processes of gossypol and fibers.

This study systematically evaluated the dynamic changes in the nutritional indicators of cottonseed hulls after fermentation by *Bacillus* and *Lactobacillus*. Using 16 S rRNA amplicon sequencing technology, a comprehensive analysis was conducted on the alpha diversity characteristics, composition structure, intergroup differences, interspecies interaction networks, and potential functional attributes of microbial communities. Furthermore, the action mechanisms of the different microorganisms in the fermentation process were revealed. This study systematically compared the regulatory mechanisms of *Bacillus* and *Lactobacillus* on nutritional indicators and microbial communities during solid-state fermentation of cottonseed hulls. High-throughput analysis was combined to reveal the phased synergistic effects of *Bacillus* and *Lactobacillus*, and a staged inoculation strategy based on changes in bacterial communities, providing both theoretical and practical innovations for the efficient fermentation and utilization of agricultural by-products.

## Materials and methods

### Materials and strains

NB medium (10 g/L peptone, 3 g/L yeast extract, 5 g/L NaCl). MRS medium (10 g/L peptone, 8 g/L beef extract, 4 g/L yeast extract, 20 g/L g/L glucose, 2 g/L K_2_HPO_4_, 2 g/L (NH_4_)_2_HC_6_H_5_O_7_, 5 g/L NaC_2_H_3_O_2_, 0.2 g/L MgSO_4_, 0.04 g/L MnSO_4_, 1 g/L Tween 80).

The cottonseed hulls were collected from Xinjiang Changji Tianhui Agriculture Co., Ltd. *Lactiplantibacillus plantarum*, *Lentilactobacillus buchneri*, *Lacticaseibacillus casei*, *Bacillus subtilis*, *Bacillus licheniformis*, and *Priestia megaterium* were all from the Institute of Microbial Applications, Xinjiang Academy of Agricultural Sciences. The concentration of all bacteria was 1 × 10^8^ CFU/mL. The chemical reagents, including 98% H_2_SO_4_, NaOH (analytical grade, ≥ 99.0%), CuSO_4_(analytical grade, ≥ 99%), 0.1 M HCl, were purchased from Yuan Ye Biotechnology Co., Ltd (Shanghai, China).

### Experimental design

In this study, three treatment groups were utilized for fermenting cottonseed hulls. *Bacillus subtilis*, *Bacillus licheniformis*, and *Priestia megaterium* were combined to form the compound *bacillus* group (Group B) at a ratio of 1:1:1. *Lactiplantibacillus plantarum*, *Lentilactobacillus buchneri*, and *Lacticaseibacillus casei* were combined to form the complex *Lactobacillus* group (R group) at a ratio of 1:1:1. This uniform proportional design was adopted to ensure a consistent initial bacterial biomass across all test groups, thereby facilitating an objective assessment and comparison of the fermentation performance between the two composite bacterial agents on cottonseed hulls. The natural fermentation group (C group) was included, with no bacterial agents added. All treatment groups were packed in vacuum fermentation bags (23 × 30, Wenzhou, Zhejiang) and underwent a 30-day fermentation process at room temperature. Throughout the fermentation process, the moisture content of the materials was kept at 60%, and the quantity of bacterial agents added was 10% of the total weight of the materials. The fermentation effects of different treatment groups were compared to assess the influence of microbial combinations on the fermentation quality of cottonseed hulls.

Fermentation sample analysis.

Samples were collected on the 1st, 3rd, 5th, 10th, and 30th days of fermentation. The free gossypol content was determined by high-performance liquid chromatography(HPLC) [15]. The crude protein content was determined using the Kjeldahl nitrogen determination method [16]. The crude fiber content was determined using a crude fiber analyzer (Ankom, Macedon, USA). The crude fat content was determined through Soxhlet extraction [17]. DNA extraction was performed on the fermented samples using the Plant Genome DNA Kit (Tiangen Biotech, Beijing, China). The extracted DNA was forwarded to Beijing Baimaike Biotechnology Co., LTD. (Beijing, China) for high-throughput sequencing. PCR amplification was performed using the forward primer 27 F (Beijing) and the reverse primer 1492R (Beijing). The original data were generated using the Illumina HiSeq sequencing platform. A small fragment library was constructed for sequencing using the Paired-End sequencing method. NCBI Sequence Read Archive Accession PRJNA1289344 was used to store the raw sequence data for the 16 S rRNA gene.

### Data analysis

Quality control of the original data was performed using Trimmomatic 0.33 to remove low-quality sequences. Primer sequences were then removed using cutadapt 1.9.1, resulting in the acquisition of 3,523,378 high-quality sequences. The sequences were clustered at a 97.0% similarity threshold using Usearch software to generate OTUs for subsequent biostatistical analysis. Alpha and Beta diversity analyses were performed using QIIME2 2020.6. SILVA was used as the reference database, and taxonomic annotation of the feature sequences and species classification of OTUs were conducted using the naive Bayesian classifier. Venn, LefSe, and Spearman correlation network analyses were performed using R software (version 4.1.1). The genetic functions of the B group and the R group were predicted using the PICRUSt and Bugbase phenotypes. Variance analysis was performed using SPSS 27 software (IBM, New York, USA) to evaluate nutritional indicators and microbial diversity at various fermentation stages. A significance level of *P* < 0.05 was used to determine statistically significant differences.

## Results

### Variations in nutritional composition

The variation of nutritional composition is shown in Table 1. The fermentation time and microbial inoculants significantly affected the detoxification and nutritional indicators during the fermentation process of cottonseed meal (*P* < 0.001). By day 30, the pH values of Group B (*Bacillus* group) and Group R (*Lactobacillus* group) were stabilized at 4.0 ± 0.2 and 5.2 ± 0.2, respectively, indicating that microbial metabolic activity had reached a state of equilibrium. Furthermore, On day 30 of fermentation, the free gossypol (FG) content in group B and group R decreased to 26.41 g·kg^− 1^ and 39.06 g·kg^− 1^, respectively, which was significantly lower than that in the control group C (42.07 g· kg^− 1^, *P* < 0.001). During the fermentation process, the crude protein (CP) content in both the treatment groups and the control group showed no significant changes (*P*>0.05). However, the recovery amplitude of group R was the greatest on the 30th day of fermentation. This may be because *Lactobacillus* can synthesize microbial proteins through its growth and reproduction [18]. The degradation efficiency of crude fiber (CR) followed the order of B > C > R, which is consistent with the conclusion reported by Liu et al. [19] that *Bacillus* possesses excellent cellulose degradation ability due to the efficient secretion of cellulase and hemicellulase. During fermentation, the crude fat (CF) content of the three sample groups showed an upward trend, but the effect of Group B on the increase in CF was not found to be significant (*P* > 0.05). Furthermore, the increase in group B and group R was significantly lower than that in group C. This phenomenon may be attributed to the inoculated microorganisms metabolizing and utilizing some lipid substances as carbon sources [20].

**Table 1:** The changes in nutritional indicators of different treatment groups during fermentation

Item	Time(days)				
	1	3	5	10	30
Free gossypol (FG, g·kg^− 1^)					
B	135.56 ± 0.30^Aa^	108.35 ± 0.87^Ab^	85.04 ± 0.62^Ac^	37.30 ± 0.19^Ad^	26.41 ± 0.08^Ae^
R	135.48 ± 0.04^Aa^	110.06 ± 0.31^Bb^	92.22 ± 0.28^Bc^	49.76 ± 0.07^Bd^	39.06 ± 0.06^Be^
C	135.46 ± 0.53^Aa^	113.42 ± 0.18^Cb^	92.69 ± 0.41^Bc^	51.90 ± 0.11^Cd^	42.07 ± 0.14^Ce^
Crude protein (CP, %)					
B	5.05 ± 0.09^Aa^	4.88 ± 0.73^Aab^	4.59 ± 0.70^Aab^	3.98 ± 0.43^Ab^	4.77 ± 0.31^Aab^
R	5.02 ± 0.50^Aab^	4.78 ± 0.27^Aab^	4.58 ± 0.69^Aab^	4.06 ± 0.54^Ab^	5.06 ± 0.43^Aa^
C	5.02 ± 0.75^Aa^	4.89 ± 0.57^Aa^	4.70 ± 0.41^Aa^	4.35 ± 0.38^Aa^	4.63 ± 0.49^Aa^
Crude fiber (CR, %)					
B	35.91 ± 0.10^Aa^	34.39 ± 0.34^Ab^	33.89 ± 1.04^Ab^	32.57 ± 0.62^Ac^	30.53 ± 0.89^Ad^
R	36.02 ± 0.15^Aa^	35.69 ± 0.32^Aa^	35.32 ± 0.65^Aab^	34.70 ± 0.60^Bbc^	34.09 ± 0.47^Bc^
C	36.18 ± 0.06^Aa^	35.68 ± 0.36^Aab^	34.94 ± 0.55^Abc^	33.84 ± 0.80^ABd^	34.08 ± 0.47^Bcd^
Crude fat (CF, %)					
B	9.05 ± 0.04^Aa^	9.11 ± 0.33^Aa^	9.17 ± 0.49^Aa^	9.36 ± 0.58^Ba^	9.43 ± 0.64^Aa^
R	9.09 ± 0.09^Ab^	9.48 ± 0.56^Ab^	9.80 ± 0.65^Aab^	10.90 ± 0.36^Aa^	9.79 ± 0.96^Aab^
C	9.03 ± 0.11^Ac^	9.60 ± 0.42^Abc^	10.19 ± 0.56^Ab^	11.87 ± 0.67^Aa^	10.13 ± 0.34^Ab^

B and R, treatment group; C, control group. Capital letters indicate a significant difference between different treatment groups on the same sampling day (ANOVA test, *P* < 0.05). Lowercase letters indicate the marked differences between different fermentation times within the same group (ANOVA test, *P* < 0.05). *N* ≥ 3 repeats

### The diversity and similarity of bacterial communities during fermentation

**Fig. 1:**
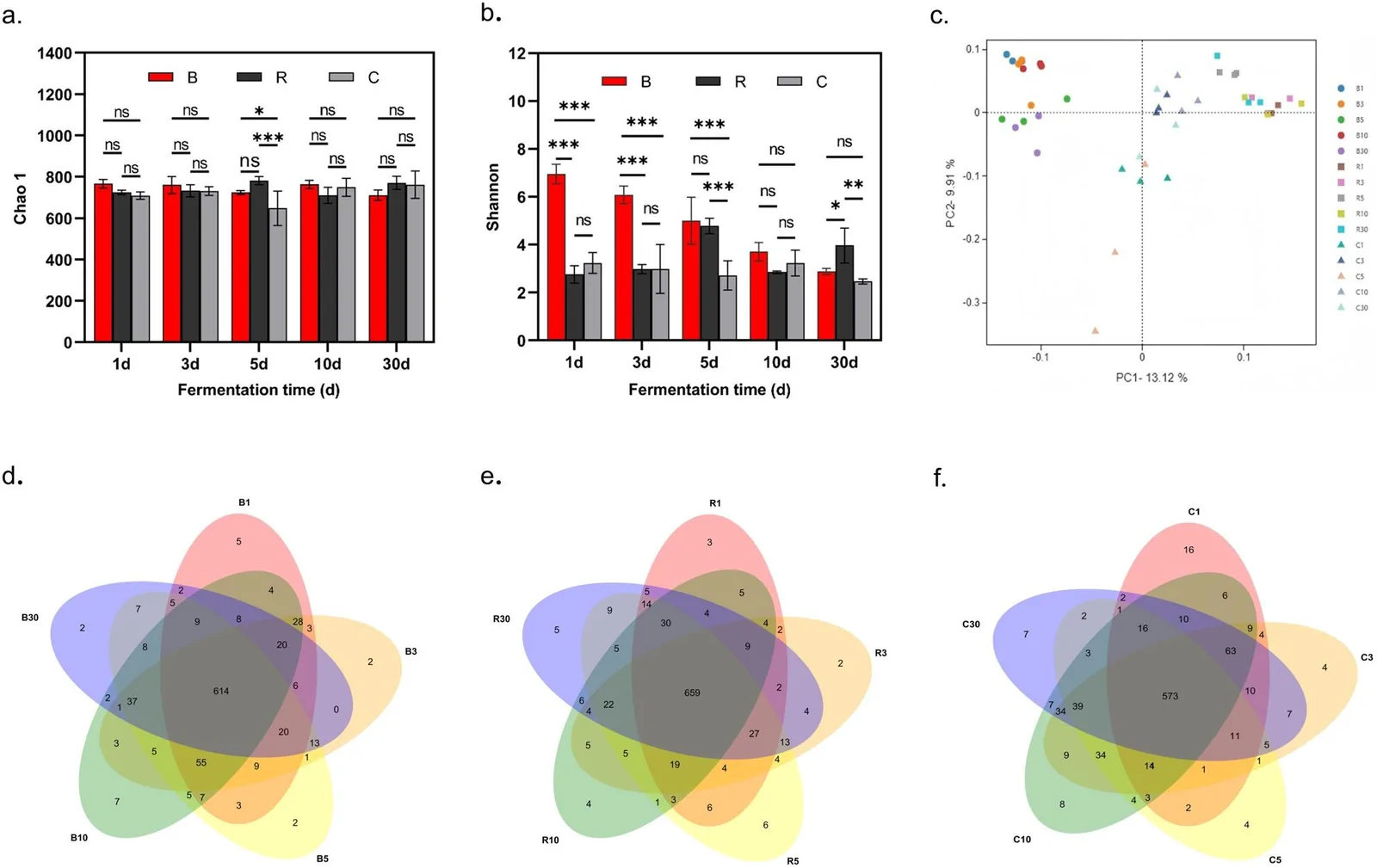
The diversity and similarity of bacterial communities under different treatments. Chao1 (**a**), Shannon (**b**), PcoA (**c**). B: the fermentation inoculated with the bacillus, R: inoculated with Lactobacillus, and C: control group. Venn analysis of bacterial OTUs during bacillus treatment (**d**), Lactobacillus treatment (**e**), and control treatment (**f**).The significant difference in different treatments at the same fermentation time was expressed with *, and ns indicated no significant difference

The Chao1 and Shannon indices represent species richness and diversity throughout fermentation. Significant changes in the microbial communities were observed under different treatment conditions. The diversity index and bacterial similarity during the fermentation period are illustrated in Fig. 1. Throughout the fermentation process, the Chao1 index for all three groups remained between 600 and 800, suggesting that the exogenous bacterial agent did not influence the species richness in each group. This could be due to the microbial community’s inherent self-regulating ability, with the exogenous bacterial agent not causing an imbalance in ecological function [21]. The Chao1 index in group B exhibited less fluctuation. In contrast, the Shannon index consistently decreased, which may be attributed to the synergistic degradation of substrates by cellulase and xylanase secreted by the *Bacillus* complex, promoting the growth of dominant strains, which leads to a decrease in community diversity [22]. The Chao1 and Shannon indices in group R fluctuated upward, peaking on the 5th day. This could be due to the acid production by *Lactobacillus* (lowering pH), which inhibits some sensitive bacteria while promoting the proliferation of acid-tolerant bacteria (such as *Acidobacterium*), thereby increasing the stability and diversity of the community [23]. The overall changes in the Chao1 and Shannon indices in group C were relatively small. On the fifth day of fermentation, the Chao1 index in group C was significantly lower than in groups B and R (*P* < 0.05), and the Shannon index in group C was also considerably lower than in treatment groups B and R (*P* < 0.001). This suggests that adding exogenous flora significantly enhances the diversity and stability of the microbial community [24]. The PCoA and Venn diagrams illustrate the similarity of species (Fig. 1). The PCoA analysis (*R* = 0.494, *P* = 0.001) revealed that the microbial community structures between groups were significantly different. The Venn analysis shows that there were 614, 659, and 573 bacterial OTUs in groups B, R, and C, respectively, throughout the entire fermentation stage, indicating high similarity in microbial communitiy composition among the three groups. Additionally, on the 1st, 3rd, 5th, and 10th days, group B exhibited 55 bacterial OTUs, higher than those in groups R (19) and C (14). This suggests that microbial community diversity in group B is more abundant. This could be due to the introduction of *Bacillus* in group B, which alters the carbon source metabolic pathways (e.g., accelerating the degradation of gossypol) [25].

### Changes in bacterial community structure during fermentation

**Fig. 2:**
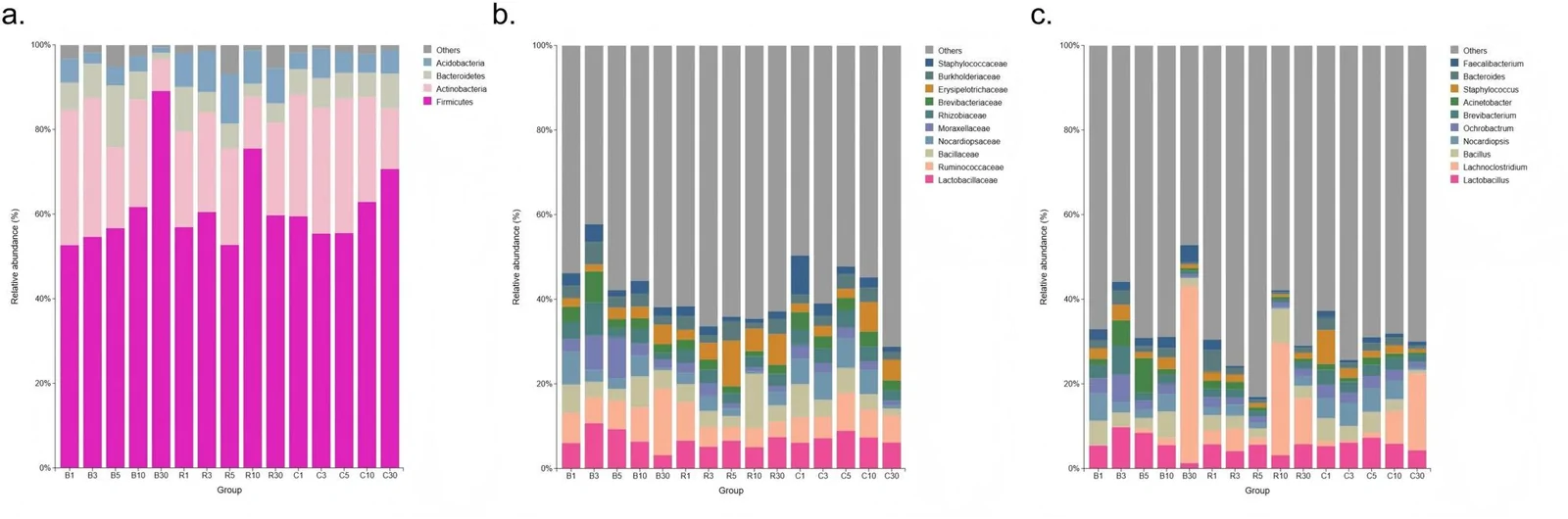
The classification of bacterial communities at different phylum, family, and genus levels during fermentation and the relative abundance of bacterial phylum (**a**), family (**b**) and genus (**c**)

During the fermentation process, the dominant bacterial phyla in groups B, R, and C were identified as *Firmicutes*, *Actinobacteria*, *Bacteroidetes*, and *Acidobacteria* (Fig. 2a). At the family level, the predominant taxa included *Lactobacillaceae*, *Ruminococcaceae*, *Bacillaceae*, *Nocardiopsaceae*, *Moraxellaceae*, *Rhizobiaceae*, *Brevibacteriaceae*, *Erysipelotrichaceae*, *Burkholderiaceae*, and *Staphylococcaceae*. As shown in Fig. 2b, the relative abundance of the typical aerobic heterotrophic family *Nocardiopsaceae* decreased markedly over time in all treatment groups. However, in the later stages of fermentation, the microbial community structure was significantly altered by the introduction of different exogenous microbial agents. On day 30, the relative abundance of *Lactobacillaceae* in the R group remained relatively high (7.44%), whereas *Ruminococcaceae* was suppressed to 3.64% (Table S1), which was significantly lower than in the C (15.75%) and B (6.39%) groups. In contrast, although the B treatment enhanced substrate degradation during the early stage, its limited acidification capacity resulted in microbial imbalance during later stages. Specifically, on day 30, *Lactobacillaceae* abundance in the B group decreased to the lowest level (3.15%), whereas *Ruminococcaceae* increased markedly (15.75%). This shift may be attributed to the accumulation of degradation products during the early stage, thereby promoting the overgrowth of anaerobic heterotrophic bacteria. At the genus level, the dominant taxa included *Lactobacillus*, *Lachnoclostridium*, *Bacillus*, *Nocardiopsis*, *Ochrobactrum*, *Brevibacterium*, *Acinetobacter*, *Staphylococcus*, *Bacteroides*, and *Faecalibacterium*. As shown in Fig. 2c, most genera in the B group increased during the initial stage and subsequently declined during the middle and late stages. Representative genera, including *Lactobacillus*, *Bacillus*, *Ochrobactrum*, *Streptococcus*, and *Faecalibacterium*, exhibited this pattern, suggesting that the B treatment strongly stimulated microbial growth during the early stage. Notably, from day 10 onward, the abundance of *Bacillus* in the R group was significantly higher than that in the B and C groups, while *Lactobacillus* remained relatively stable throughout the fermentation process, suggesting that the R treatment was more effective in maintaining microbial stability. Additionally, *Lachnoclostridium* exhibited pronounced fluctuations in the B and R groups, reaching peak abundances on days 30 and 10, respectively, with the abundance in the B group being 51.62% higher than that in the R group. This pattern may be related to the role of *Bacillus* in enhancing anaerobic conditions during cottonseed hull fermentation. At the end of fermentation, the relative abundances of *Lactobacillus*, *Bacillus*, *Nocardiopsis*, *Ochrobactrum*, *Brevibacterium*, *Acinetobacter*, *Staphylococcus*, and *Bacteroides* in the R group were generally higher than those in the B and C groups, further confirming that the R treatment more effectively optimized the microbial community structure. 

### Gene function prediction

**Fig. 3:**
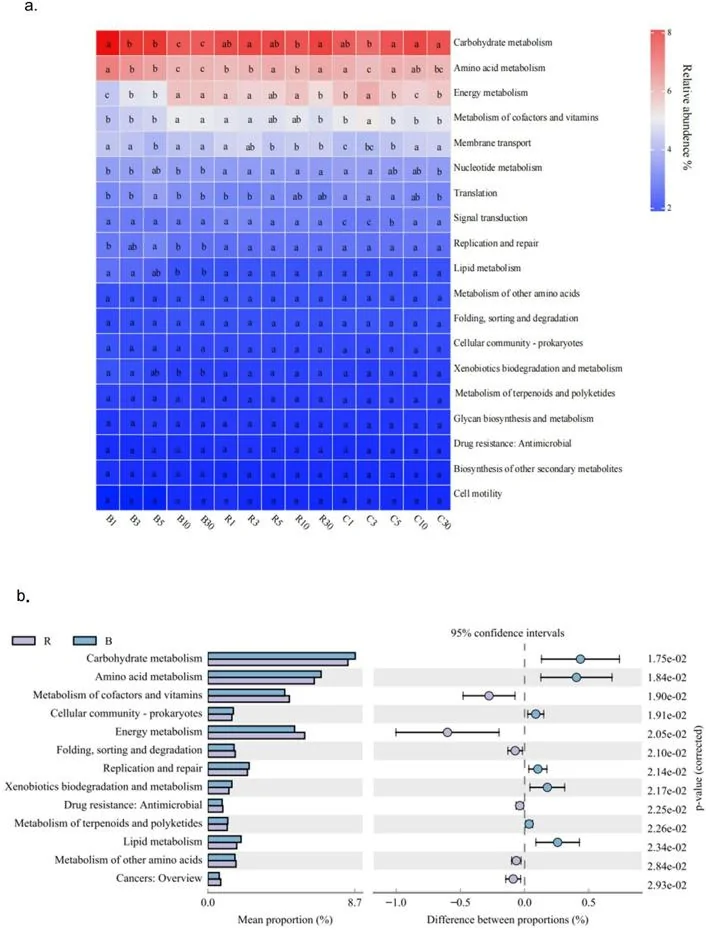
The differentiation of functional gene prediction between different treatment groups. Different lowercase letters represent significant differences at *P* < 0.05 based on the analysis of variance in different fermentation times of the same treatment (**a**). KEGG metabolic pathways at the second level (**b**)

The prediction of PICRUST gene function indicated that the predicted metabolic potential among groups B, R, and C was highly similar (Table S2). Metabolic function (78%-79%) represents the most significant functional category in cottonseed hull fermentation, suggesting that microbial communities under various inoculation treatments possess strong substrate utilization potential, with core functions centered around the decomposition and transformation of cottonseed hulls [26]. With the extension of fermentation time, significant changes were observed in functions including carbohydrate metabolism, amino acid metabolism, energy metabolism, cofactor and vitamin metabolism, and membrane transport across different treatment groups (Fig. 3a). Notably, in group B, carbohydrate metabolism and amino acid metabolism decreased significantly, whereas energy metabolism, along with cofactor and vitamin metabolism, gradually increased. Meanwhile, carbohydrate metabolism in groups R and C gradually increased, while amino acid metabolism in these groups exhibited fluctuating upward and downward trends, respectively. Furthermore, energy metabolism, as well as the metabolism of cofactors and vitamins in group R, gradually decreased, whereas that in group C remained largely stable. Additionally, membrane conduction and signal conduction in group C increased significantly, whereas those in groups B and R remained largely unchanged. These findings suggest that the addition of microbial agents may have enhanced the stability of the microbial community. Figure 3b (second level) further analyzes the differences in metabolic pathways between groups B and R and identifies 13 pathways with significant differences (*P* < 0.05). Regarding the overall metabolic pathway, group B exhibits higher activity in carbohydrate and amino acid metabolism, while group R shows significant advantages in energy metabolism, as well as in the metabolism of cofactors and vitamins.

### Correlation network analysis

**Fig. 4:**
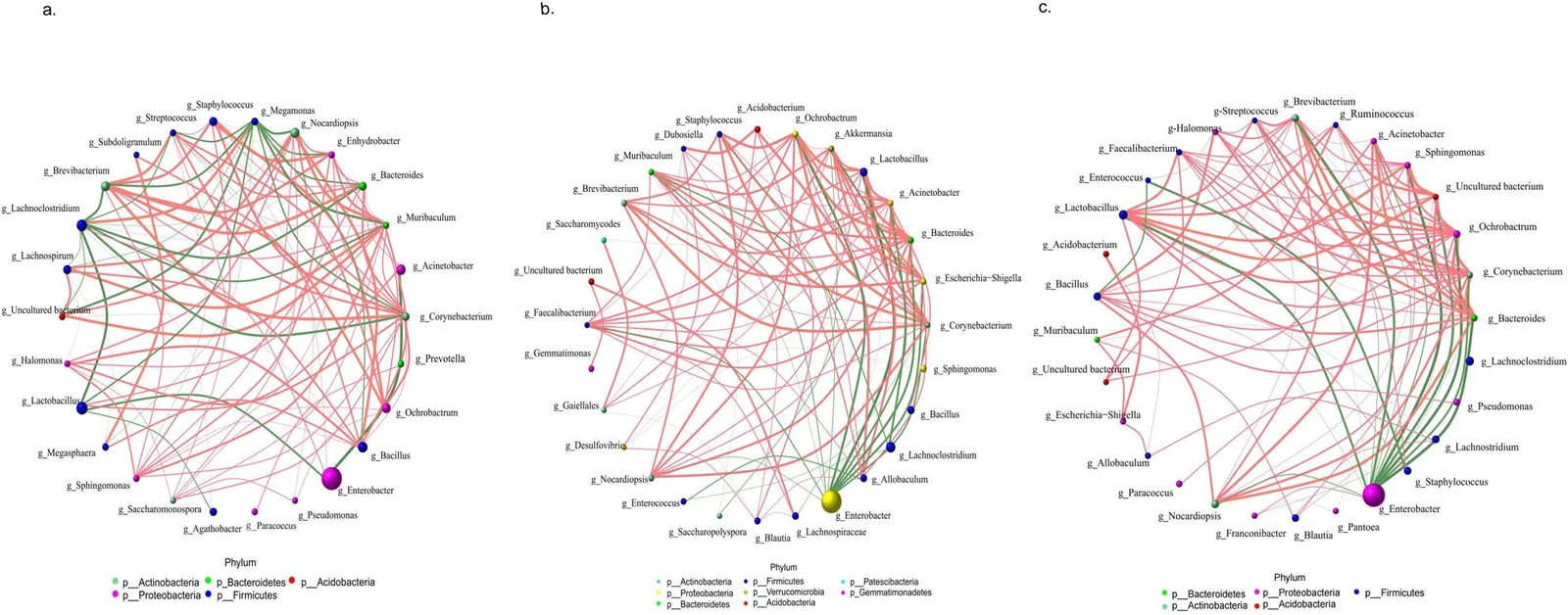
Bacterial correlation network in different treatment groups during fermentation. Co-occurrence networks of the B group ( **a**), the R group (**b**), and the control group (**c**) are shown. The red lines represent significantly positive (*r* > 0.6) interrelationships, and the green lines represent negative (*r* < -0.6) interrelationships. The node size represents the degree, and the line width represents the correlation’s strength

During the fermentation of cottonseed hulls, interaction relationships among different microorganisms exist. The complexity and diversity of microbial communities can be further examined through correlation network analysis [27]. The correlation network diagram (Fig. 4) illustrates that the average degree of the bacterial network treated in group B was 7.70% and 11.45% higher than that in group R and group C, respectively. Indicating that inoculation with microbial agents increased the complexity of the bacterial network, and the complexity of the network in group B was higher than that in group R. The positive bonds in the bacterial networks of group B, R, and C exceeded the negative bonds, and all were positively correlated. In addition, the number of positive bonds in group C was 82, in group R it was 80, and in group B it was 73, suggesting that *Bacillus* may weaken the symbiotic interaction between microorganisms. Furthermore, eight genera positively correlated with *Bacillus* in the control group, an increase of 1 in group B, and a decrease of 8 in group R. The number of genera positively correlated with *Lactobacillus* was 10 in the control group, decreased by 1 in group R, and 8 in group B (Table S3). This may be due to the clear competitive interaction between *Bacillus* and *Lactobacillus* in the cottonseed husk fermentation system. Therefore, direct competition should be avoided when designing compound bacterial agents by optimizing the inoculation ratio or timing.

### Correlations of bacterial biomarkers and nutritional factors during the fermentation

**Fig. 5:**
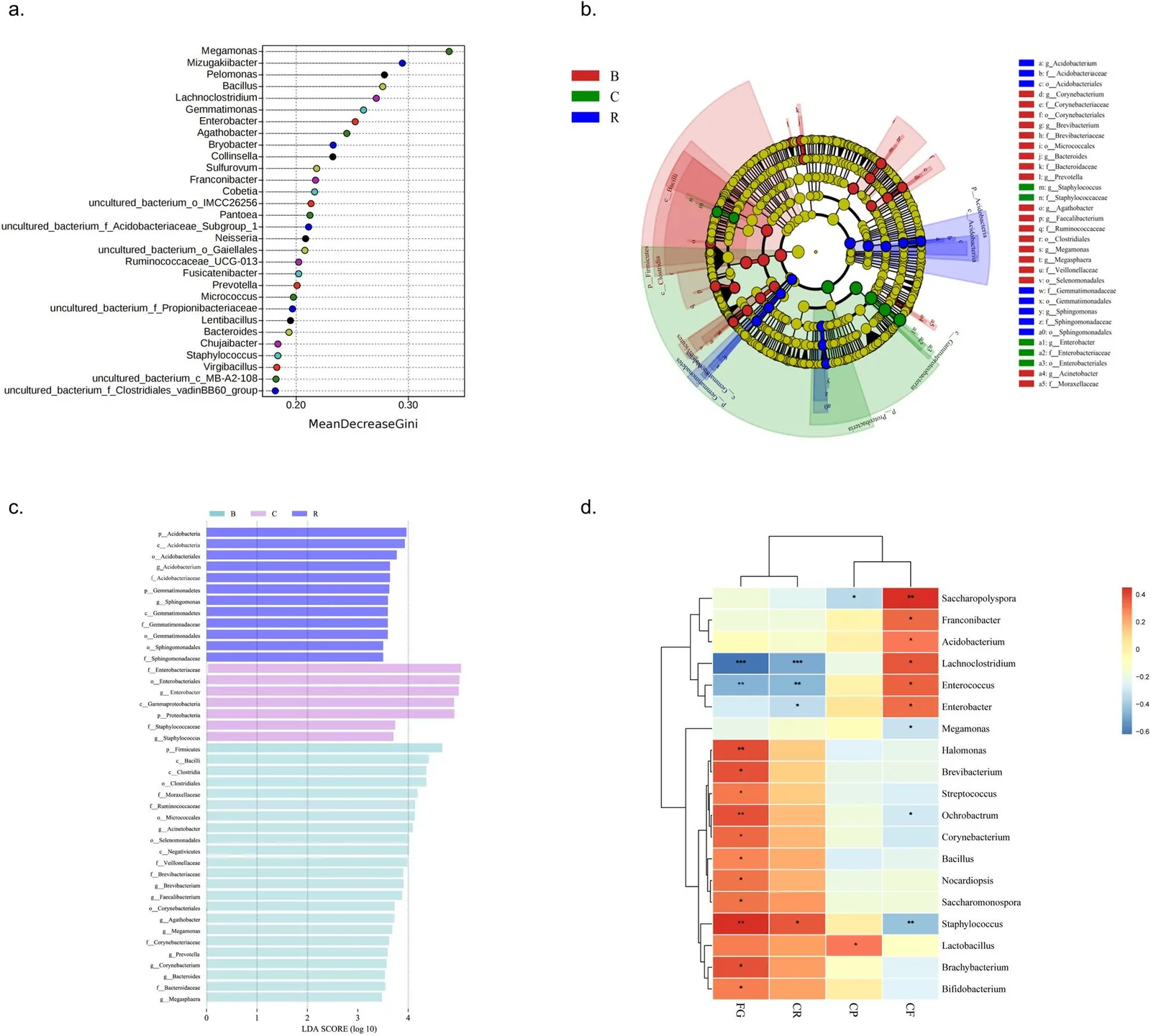
Random Forest Model Analysis (**a**), Lefse analysis (**b**), LDA analysis (**c**) of bacterial communities, and the heatmap of the correlation between nutritional factors and microorganisms (**d**). *P<0.05, **P<0.01, ***P<0.001

The top 30 microbial markers related to feed fermentation effects were identified through the random forest model (Fig. 5a). The microbial genera *Megamonas*, *Pelomonas*, *Bacillus*, *Lachnoclostridium*, *Enterobacter*, *Agathobacter*, *Bryobacter*, *Collinsella*, *Pantoea*, *Fusicatenibacter*, *Prevotella*, *Lentibacillus*, *Bacteroides*, and *Virgibacillus* in the feed industry exhibit significant fermentation potential. *Megamonas*, *Lachnoclostridium*, *Enterobacter*, *Agathobacter*, and *Pantoea* are capable of breaking down carbohydrates and producing organic acids, which help inhibit pathogenic bacteria produced during the feed fermentation process [28–31]. *Pelomonas*, *Bacillus*, *Bryobacter*, *Collinsella*, *Fusicatenibacter*, *Prevotella*, *Lentibacillus*, *Bacteroides*, and *Virgibacillus* all exhibit strong abilities to decompose organic matter and participate in the breakdown of cellulose and other complex organic compounds [32–35]. Additionally, LefSe analysis revealed specific bacterial biomarkers in different treatment groups during the fermentation process of cottonseed shells. As shown in Fig. 5b and c (LDA = 3.5), at the Phylum level, the biomarker for group B was *Firmicutes*, while the biomarkers for group R included *Acidobacteria* and *Gemmatimonadetes*, and that for group C was *Proteobacteria*. At the genus level, the biomarkers for Group B include *Acinetobacter*, *Brevibacterium*, *Faecalibacterium*, *Agathobacter*, *Megamonas*, *Prevotella*, *Corynebacterium*,* Bacteroides*, and *Megasphaera. Acidobacterium* and *Sphingomonas* in Group R, and *Enterobacter*, and *Staphylococcus* in Group C. Additionally, to reveal the functional connections between microorganisms and nutrient transformation, a correlation analysis between microbial communities and nutrient transformation during the fermentation process was conducted (Fig. 5d). A total of 19 bacterial genera were found to be significantly correlated with nutritional indicators (*P* < 0.05). The genera positively correlated with FG degradation include *Halomonas*, *Brevibacterium*, *Streptococcus*, *Ochrobacterium*, *Corynebacterium*, *Bacillus*, and *Nocardiopsis*. The genera *Lachnoclostridium* and *Enterobacter*, which show a significant negative correlation, include *Saccharomonospora*, *Staphylococcus*, *Brachybacterium*, and *Bifidobacterium*. The genus *Staphylococcus* is significantly positively correlated with CR degradation, whereas the genera *Lachnoclostridium*, *Enterococcus*, and *Enterobacter* are significantly negatively correlated. The genus *Lactobacillus* was significantly positively correlated with an increase in CP, whereas the genus *Saccharopolyspora* was significantly negatively correlated. The genera significantly positively correlated with an increase in CF include *Saccharopolyspora*, *Franconibacter*, *Acidobacterium*, *Lachnoclostridium*, *Enterococcus*, and *Enterobacter*. The genera that show significant negative correlations include *Ochrobacterium* and *Staphylococcus*. These results indicate that Group B has an advantage in transforming specific nutrients, such as gossypol and fiber degradation. Meanwhile, some microorganisms in Group R may play a more significant role in protein and lipid synthesis.

## Discussion

### Effects of different treatment groups on microbial diversity and composition during cottonseed hull fermentation

The analysis of microbial diversity results indicates that the microbial community composition among different treatment groups is highly similar. Furthermore, Significant differences in microbial diversity were observed. As fermentation time progressed, microbial diversity in group B gradually decreased, while that in group R exhibited a fluctuating upward trend, and that in group C remained largely stable. However, overall, microbial diversity in group B was greater. This may be due to *Bacillus*’s ability to rapidly regulate complex microecosystems, while *Lactobacillus* focuses more on maintaining microbial community stability [36, 37]. *Bacillus* can rapidly decompose organic matter during the early stages of fermentation, a finding related to Zhou et al.‘s [38]discovery that *Bacillus* enhances the functional stability of organic waste during fermentation. Furthermore, in the latter stage of fermentation, the relative abundance of most bacterial genera in Group R was significantly higher than that in Groups B and C (Table S1). This observation suggests that the predominant position of *Lactobacillus*, which may be closely related to the stability of its metabolic activities and its acid-producing capacity, reflects the dynamic interactions within the microbial community during fermentation [39].

### Metabolic differences between *Lactobacillus* and *Bacillus* during the fermentation of cottonseed hulls

The metabolic differences between *Bacillus* and *Lactobacillus* indicate that *Bacillus* possesses a more diverse hydrolase system. For instance, *Bacillus* is capable of secreting a variety of extracellular hydrolases (such as cellulase, pectinase, and protease) [40, 41], which efficiently degrade the complex polysaccharide and protein matrices in cottonseed shells, thereby initiating the degradation and metabolic processes of macromolecular substrates during the early stage of fermentation and establishing the initial metabolic network [42, 43]. Concurrently, *Lactobacillus* are able to produce lactic acid and a range of functional metabolites (such as potential antibacterial substances and metabolic cofactors) via efficient carbohydrate metabolic pathways [44]. These metabolites not only rapidly reduce system pH, thereby inhibiting the growth of undesirable microorganisms, but also enhance the overall tolerance and stability of the fermentation environment, thereby providing a critical biochemical basis for the continuous and stable progression of fermentation [45]. Based on this functional differentiation, it can be inferred that the two types of microorganisms exhibit a stage-dependent cooperative interaction during the fermentation process. During the early stage of fermentation, *Bacillus* predominantly drives substrate degradation through extracellular enzyme secretion, thereby supplying readily utilizable small-molecule substrates for subsequent metabolism [46]; whereas during the later stage, *Lactobacillus* becomes dominant through the accumulation of functional metabolites, thereby playing a key role in regulating pH, inhibiting competing microorganisms, and maintaining microecological stability [47, 48]. This sequential functional division and cooperative mechanism is likely a key driving factor underlying the efficiency and stability of the fermentation system. However, several limitations are acknowledged in this study. Firstly, although metabolic differences between *Bacillus* and *Lactobacillus* at various fermentation stages have been observed, the specific metabolic pathways and required to perform more comprehensive metabolomic analyses in order to identify the key enzymes and functional substances that play decisive roles in the fermentation process. Secondly, during the fermentation process, the metabolic activity of the strains is influenced by various factors (such as substrate type and environmental conditions); therefore, the generalizability of the existing results across different fermentation systems still requires further verification.

### The influence of different treatment methods on the microbial network of cottonseed hulls

The introduction of *Bacillus* enhances the complexity of the bacterial network, but it reduces bacterial cooperativeness. This phenomenon may be attributed to the competitive role *Bacillus* assumes during the fermentation process. Its strong substrate degradation capacity and tolerance allow it to compete with other microorganisms, thereby disrupting the original symbiotic relationship. *Bacillus* and *Lactobacillus* may particularly engage in a competitive relationship within the fermentation system. *Bacillus* may inhibit the growth and metabolism of *Lactobacillus* by occupying ecological niches, as supported by existing studies. Du et al. [49]stated that the complex competitive and symbiotic relationships within microbial communities significantly impact system function and stability. Therefore, to reduce resource competition and ecological niche overlap among core functional microbial communities, it is essential to systematically optimize inoculation strategies during the development of composite microbial consortia and the design of fermentation processes. A preferred approach involves the implementation of a staged inoculation strategy. During the initial stage of fermentation, *bacillus* are first introduced to exploit their robust aerobic metabolic capacity, enabling rapid depletion of dissolved oxygen and promoting the initial degradation of complex macromolecular substrates (e.g., proteins and polysaccharides), thereby generating readily utilizable low-molecular-weight nutrients for subsequent microbial growth [50]. As the fermentation system progressively transitions to an anaerobic or facultative anaerobic environment and fermentable carbon sources accumulate, *Lactobacillus* is subsequently introduced to maximize its acidogenic metabolic activity under low-oxygen conditions [51].This staged regulatory strategy facilitates ecological niche differentiation of microbial communities along the temporal axis, reduces direct competitive interactions, and promotes metabolic division of labor and functional complementarity, thereby enhancing the overall efficiency and stability of the fermentation system [52]. In simultaneous fermentation systems, the community structure should be modulated by adjusting the initial inoculation ratios. Specifically, the initial abundance of spore-forming bacteria should be reduced, while the proportion of *lactobacillus* should be increased to attenuate the competitive dominance of the former during the early fermentation stage and prevent excessive occupation of substrates and ecological niches [53]. This approach facilitates the progression of the microbial community toward a dynamically balanced symbiotic system, thereby maximizing synergistic interactions among microbial populations [54].

### The correlation between biomarkers and nutritional enhancement

The core microbial markers of different treatment groups during cottonseed hull fermentation, along with their correlation with nutrient transformation, were identified using random forest and LefSe analysis. The results indicated that genera such as *Bacillus*, and *Lachnoclostridium* played a key role in the decomposition of organic matter and the production of organic acids. This helps to inhibit pathogenic bacteria and promote the utilization of fibers and proteins [55]. Correlation analysis between the nutritional components of cottonseed hulls and bacterial genera revealed pronounced metabolic functional differentiation among treatment groups. Group B exhibited enhanced capacities for gossypol degradation and fiber decomposition but showed no significant promotion of crude fat or protein synthesis. This pattern may be attributed to the metabolic traits of *Bacillus*, whose members secrete high levels of cellulases, hemicellulases, and gossypol-degrading enzymes (e.g., peroxidases and proteases), facilitating efficient disruption of the lignocellulosic matrix and detoxification of gossypol [56, 57]. However, metabolic activity in this community is largely directed toward carbon utilization and extracellular enzyme production, while neutral to mildly alkaline conditions may exacerbate nitrogen volatilization, thereby constraining CF accumulation and protein synthesis [58]. In contrast, rapid acidification in Group R markedly reduced system pH, thereby suppressing spoilage microorganisms and endogenous protease activity, and consequently limiting protein degradation and nitrogen loss [59]. Accordingly, Group R exhibited a pronounced advantage in the accumulation of CP and CF. Although the net increase in CF in Group R was lower than that in Group C (Table 1), the final levels remained significantly above the initial baseline, indicating that exogenous microbial inoculation effectively promotes crude fat biosynthesis and accumulation. The lower net increase is more likely attributable to metabolic flux redistribution, rather than diminished biosynthetic capacity [60]. However, the current results are primarily based on correlation analysis. It is still necessary to integrate metabolomics and functional gene verification to clarify causal relationships and further explore the utilization of these key microorganisms to optimize the fermentation process and enhance the nutritional value of feed. Future studies will focus on multi-omics integration, incorporating metabolomics, functional gene profiling, and targeted functional validation experiments. A systematic analysis of the roles of key microorganisms in the fermentation process will be conducted at both the metabolic pathway and molecular mechanism levels. By constructing an integrated framework linking microbial composition, functional metabolites, and gene characterization, clearer causal relationships are expected to be established, thereby providing a more robust theoretical basis for functional strain screening, fermentation process optimization, and feed nutritional enhancement.

## Conclusion

*Bacillus* and *Lactobacillus* exert significant differential regulatory effects on the nutritional enhancement and microbial community succession during the solid-state fermentation of cottonseed hulls. *Bacillus* dominates fiber degradation and increases the complexity of the microbial interaction network, while *Lactobacillus* suppresses excessive protein decomposition through acid production and optimizes community stability. A competitive interaction between *Bacillus* and *Lactobacillus* was revealed by correlation network analysis. These findings suggest that fermentation efficiency could be enhanced by implementing a staged inoculation strategy or by optimizing the inoculation ratio of the two bacterial groups. and identifies key biomarkers significantly associated with FG, FR degradation, CP, and CF. Investigation of the distinct mechanisms underlying the roles of *Bacillus* and *Lactobacillus* in the solid-state fermentation of cottonseed hulls can enhance fermentation efficiency and stability, while providing insights into the resource utilization of agricultural by-products. Solid-state fermentation has been shown to improve the utilization of agricultural waste and mitigate environmental challenges. Future research should focus on elucidating the metabolic pathways of *Bacillus* and *Lactobacillus* to better understand their interactions and distinctions across microbial genera.

## Supplementary information

Supplementary Material 1.

## Data Availability

The DNA sequencing datasets generated and/or analyzed during the current study are available in the [NCBI] repository, [BioProject PRJNA1289344]

## Abbreviations

NB: Nutrient Broth (medium)
MRS: De Man, Rogosa, and Sharpe (medium)
FG: Free goosypol
CP: Crude protein
CR: Crude fiber
CF: Crude fat
