# Supplementary material for: Comparative evaluation of Bacillus and Lactobacillus in cottonseed hull fermentation
Source: BMC Microbiol. 2026 Aug 5;26:943. doi: 10.1186/s12866-026-05090-9 (PMC13726030; doi:10.1186/s12866-026-05090-9)
Supplement: Supplementary file 1 [file 12866_2026_5090_MOESM1_ESM.docx]

**Supplementary information**

**Comparative evaluation of Bacillus and Lactobacillus in cottonseed hull fermentation**

Kunyi Li^1,2^, Yang Li^1^, Xin Huang^1^, Huaibing Yao^1^, Gulinigaer Aiyisirehong^1^, Nuerbiya Aibaidula^1^ ,Weidong Cui^1, *^, Min Hou^1, *^

^1^Xinjiang Laboratory of Special Environmental Microbiology, Institute of Microbiology, Xinjiang Academy of Agricultural Sciences, Urumqi 830091, China.

^2^Key Laboratory of Biological Resources and Genetic Engineering, College of Life Science and Technology, Xinjiang University, Urumqi 830017, China

**Table S1.** The relative abundance of bacterial class and genus during fermentation

| Bacterial community during fermentation | | Day 1 | | |  | Day3 | | |  | Day 5 | | |  | Day 10 | | |  | Day 30 | | |
| --- | --- | --- | --- | --- | --- | --- | --- | --- | --- | --- | --- | --- | --- | --- | --- | --- | --- | --- | --- | --- |
|  |  | B | R | C |  | B | R | C |  | B | R | C |  | B | R | C |  | B | R | C |
| Bacterial  Phylum  (%) | Firmicutes | 52.69c | 57.01b | 59.52a |  | 54.67b | 60.57a | 55.44b |  | 56.7a | 52.75b | 55.49a |  | 61.75b | 75.54a | 62.92b |  | 89.16a | 59.76c | 70.65b |
|  | Actinobacteria | 31.96c | 22.59a | 28.73b |  | 32.85a | 23.5c | 29.7b |  | 19.14c | 22.73b | 31.81a |  | 25.37a | 12.27b | 24.78a |  | 7.52c | 21.84a | 14.26b |
|  | Bacteroidetes | 6.48b | 10.59a | 6.09b |  | 8.07a | 4.82b | 7a |  | 14.61a | 5.95b | 6.06b |  | 6.69a | 3.09b | 5.78a |  | 1.5c | 4.64b | 8.29a |
|  | Acidobacteria | 5.64b | 7.96a | 3.84c |  | 2.6c | 9.73a | 6.92b |  | 4.47b | 11.84a | 4.92b |  | 3.65b | 7.9a | 4.49b |  | 1.28c | 8.31a | 5.64b |
|  | Others | 3.23a | 1.85b | 1.82b |  | 1.81a | 1.38a | 0.93a |  | 5.08b | 6.73a | 1.72c |  | 2.54a | 1.2b | 2.03a |  | 0.53b | 5.45a | 1.16b |
| Bacterial family  (%) | Lactobacillaceae | 6.09a | 6.59a | 6.13a |  | 10.74a | 5.19c | 7.18b |  | 9.35a | 6.63b | 8.94a |  | 6.39b | 5.11c | 7.39a |  | 3.15c | 7.44a | 6.22b |
|  | Ruminococcaceae | 7.11b | 9.09a | 6.01b |  | 6.12a | 4.68b | 5.03b |  | 6.75b | 3.29c | 9.04a |  | 8.16a | 4.47c | 6.62b |  | 15.75a | 3.64c | 6.39b |
|  | Bacillaceae | 6.69a | 4.29b | 7.86a |  | 3.68a | 3.83a | 4.07a |  | 2.75b | 2.54b | 5.86a |  | 7.32b | 12.87a | 3.66c |  | 4.41a | 3.85a | 1.55b |
|  | Nocardiopsaceae | 7.79a | 2.64c | 5.96b |  | 2.81b | 3.4b | 6.52a |  | 2.46b | 1.84b | 7.05a |  | 4.94a | 0.53b | 5.67a |  | 0.47b | 3.08a | 0.86b |
|  | Moraxellaceae | 2.94a | 2.4a | 3.17a |  | 8.12a | 3.06b | 2.09b |  | 9.69a | 1.05c | 2.58b |  | 2.8a | 0.96b | 2.07a |  | 2.05a | 1.49ab | 1.04b |
|  | Rhizobiaceae | 4.1a | 3.06b | 3.83ab |  | 7.78a | 3.23b | 3.47b |  | 2.26b | 2.46b | 3.97a |  | 3.36a | 2.55ab | 3.49a |  | 1.73ab | 2.87a | 2.42a |
|  | Brevibacteriaceae | 3.56a | 2.36b | 4.07a |  | 7.38a | 2.38b | 2.93b |  | 2.1ab | 1.67b | 2.98a |  | 2.56ab | 1.28c | 3.55a |  | 2.05a | 2.16a | 2.42a |
|  | Erysipelotrichaceae | 2a | 2.4a | 1.97a |  | 1.63b | 4a | 2.45b |  | 2.66b | 10.79a | 2.18b |  | 2.75c | 5.32b | 6.97a |  | 4.57b | 7.24a | 4.84b |
|  | Burkholderiaceae | 2.94a | 3.2a | 2.14ab |  | 5.34a | 1.79b | 2.33b |  | 2.62b | 4.56a | 3.38b |  | 2.94a | 1.28b | 3.37a |  | 2.05b | 3.59a | 1.9b |
|  | Staphylococcaceae | 3b | 2.36b | 9.26a |  | 4.19a | 2.13b | 2.99b |  | 1.56a | 1.1a | 1.89a |  | 3.22a | 1.06b | 2.48a |  | 2.05a | 1.75a | 1.21a |
|  | Others | 53.77b | 61.61a | 49.61c |  | 42.2c | 66.3a | 60.92b |  | 57.8b | 64.06a | 52.14c |  | 55.57b | 64.57a | 54.73b |  | 61.73b | 62.89b | 71.16a |
| Bacterial genues  (%) | Lactobacillus | 5.88a | 6.24a | 5.74a |  | 10.34a | 4.74c | 6.67b |  | 8.89a | 6.60b | 7.95a |  | 5.91a | 3.48b | 6.54a |  | 1.36c | 6.50a | 4.68b |
|  | Lachnoclostridium | 0.11c | 3.61a | 1.39b |  | 0.22b | 5.99a | 0.67b |  | 1.18a | 1.94a | 1.39a |  | 1.95c | 28.88a | 8.49b |  | 43.79a | 12.06c | 19.35b |
|  | Bacillus | 6.16a | 3.88b | 5.74a |  | 3.47a | 3.42a | 3.56a |  | 2.58b | 2.55b | 5.27a |  | 6.60b | 8.71a | 3.11c |  | 1.93b | 3.27a | 0.91c |
|  | Nocardiopsis | 6.98a | 2.09c | 5.13b |  | 2.55 b | 2.95b | 5.73a |  | 2.21b | 1.58b | 5.91a |  | 4.30a | 0.36b | 4.91a |  | 0.21b | 2.51a | 0.52b |
|  | Ochrobactrum | 3.76a | 2.50ab | 3.32a |  | 6.97a | 1.94b | 2.78b |  | 2.04b | 1.63b | 3.23a |  | 2.56a | 1.23b | 2.74a |  | 0.64b | 1.97a | 1.43a |
|  | Brevibacterium | 3.45a | 2.23b | 3.82a |  | 7.19a | 2.18b | 2.72b |  | 2.00 a | 1.67a | 2.77a |  | 2.39a | 0.87b | 3.16a |  | 0.93b | 1.88a | 1.82a |
|  | Acinetobacter | 1.49a | 2.05a | 1.54a |  | 6.42a | 2.02b | 1.06b |  | 8.63a | 0.92b | 1.94b |  | 1.43a | 0.58a | 1.21a |  | 0.71a | 0.81a | 0.65a |
|  | Staphylococcus | 2.68b | 2.18b | 8.64a |  | 3.84a | 1.94b | 2.39b |  | 1.46a | 1.10a | 1.66a |  | 2.91a | 0.65b | 2.16a |  | 0.93a | 1.52a | 0.91a |
|  | Bacteroides | 2.04c | 5.66a | 3.20b |  | 3.51a | 1.87b | 1.39b |  | 1.57a | 0.97ab | 2.03a |  | 2.39a | 0.51b | 2.11a |  | 0.57a | 1.39a | 0.91a |
|  | Faecalibacterium | 2.78a | 2.63a | 1.70n |  | 2.26a | 0.47b | 0.78b |  | 1.98a | 0.79ab | 1.48a |  | 2.82a | 0.51b | 1.00b |  | 4.21a | 0.49b | 0.91b |
|  | Others | 64.67b | 66.93a | 59.79c |  | 53.23a | 72.47b | 72.26b |  | 67.46b | 80.24a | 66.36b |  | 66.72a | 54.21c | 64.56b |  | 44.71b | 67.58a | 67.92a |

B:bacillus treatment; R:lactobacillus treatment; C: control treatment. Different lowercase letters represent significant differences at p < 0.05 based on the analysis of variance in different treatment of the same fermentation time.

**Table S2**. The relative abundance of the predicted KEGG Orthologs functional profiles (KEGG level 1) during the composting process

| Function |  | Day1 | | |  | Day3 | | |  | Day5 | | |  | Day10 | | |  | Day30 | | |
| --- | --- | --- | --- | --- | --- | --- | --- | --- | --- | --- | --- | --- | --- | --- | --- | --- | --- | --- | --- | --- |
|  |  | B | R | C |  | B | R | C |  | B | R | C |  | B | R | C |  | B | R | C |
| Metabolism % |  | 79.03a | 78.28a | 78.76a |  | 78.85ab | 78.30a | 78.63a |  | 78.57ab | 78.41a | 78.53a |  | 78.52ab | 78.22a | 78.80a |  | 77.89b | 78.17a | 77.95a |
| Genetic Information Processing % |  | 6.98a | 7.01a | 7.21a |  | 7.06a | 7.10a | 7.26a |  | 7.44a | 7.17a | 7.17a |  | 7.19a | 7.07a | 7.23a |  | 6.98a | 6.98a | 6.97a |
| Environmental Information Processing % |  | 6.98a | 7.00a | 6.65a |  | 6.75a | 6.91a | 6.57a |  | 6.59a | 6.80a | 6.74a |  | 6.72a | 6.97a | 6.73a |  | 7.32a | 7.17a | 7.10a |
| Human Diseases % |  | 3.02a | 3.11a | 2.97a |  | 3.06a | 3.05a | 3.08a |  | 3.04a | 3.03a | 3.09a |  | 3.04a | 3.02a | 3.05a |  | 2.97a | 2.81a | 3.03a |
| Cellular Processing % |  | 3.05a | 2.8a7 | 2.57a |  | 2.87a | 2.68a | 2.53a |  | 2.85a | 2.73a | 2.52a |  | 2.65a | 2.71 | 2.61a |  | 2.84a | 2.88a | 2.80a |
| Orangismal Systems % |  | 1.53a | 1.80a | 1.80a |  | 1.65a | 1.90a | 1.92a |  | 1.68a | 1.84a | 1.83a |  | 1.87a | 1.91a | 1.90a |  | 1.97a | 1.92a | 1.82a |

Values are means for triplicate replicates. Different lowercase letters represent significant differences at p < 0.05 based on the analysis of variance in different composting times of the same treatment. B: bacillus treatment; R: lactobacillus treatment; C: control treatment.

**Table S3.** Microbial network parameters of the fermentation

| Network parameters | B | R | C |
| --- | --- | --- | --- |
| Nodes | 26 | 28 | 29 |
| Positive links/Total links | 73/100 | 80/100 | 82/100 |
| Negative links/Total links | 27/100 | 20/100 | 18/100 |
| Positive links/Negative links | 73/27 | 80/20 | 82/18 |
| Average degree | 7.69 | 7.14 | 6.90 |
| Average clustering coefficient | 0.60 | 0.56 | 0.54 |
| Average betweenness centrality | 0.03 | 0.05 | 0.05 |
| Average closeness centrality | 0.48 | 0.47 | 0.37 |
| Genus positively correlated with Bacillus | 9 | 1 | 8 |
| Genus positively correlated with Lactobacillus | 2 | 9 | 10 |
